# Intraocular fluid biomarkers (liquid biopsy) in human diabetic retinopathy

**DOI:** 10.1007/s00417-021-05285-y

**Published:** 2021-07-03

**Authors:** Edoardo Midena, Luisa Frizziero, Giulia Midena, Elisabetta Pilotto

**Affiliations:** 1 Department of Neuroscience–Ophthalmology, University of Padova, Padova, Italy; 2 IRCCS-Fondazione Bietti, Rome, Italy

**Keywords:** Liquid biopsy, Aqueous humor, Vitreous, Proteomics, Metabolomics, Diabetes, Diabetic retinopathy, Diabetic macular edema

## Abstract

**Purpose:**

This article aims to review the impact of detecting and quantifying intraocular biomarkers (liquid biopsy) in both aqueous and vitreous humor in eyes of people affected by diabetes mellitus.

**Methods:**

This is a detailed review about aqueous and/or vitreous humor sampling in human diabetic eyes for proteomic and/or metabolomic analysis contributing to the understanding of the pathophysiology and treatment effects of diabetic retinopathy.

**Results:**

Aqueous and vitreous humor molecular biomarkers proved to be directly correlated to each other and valuable to study retinal conditions. Moreover, proteomic and metabolomic analysis showed that the biomarkers of neuroinflammation, neurodegeneration, and vasculopathy are detectable in intraocular fluids and that their concentration changes in different stages of disease, and in response to treatment of all diabetic retinopathy aspects, mainly diabetic macular edema and proliferative retinopathy.

**Conclusions:**

Liquid biopsy offers the possibility to improve our knowledge of intraocular eye disease induced by diabetes mellitus. The exact quantification of intraocular biomarkers contributes to the precision medicine approach even in the diabetic retinopathy scenario. The diffusion of this approach should be encouraged to have quantifiable information directly from the human model, which may be coupled with imaging data. 
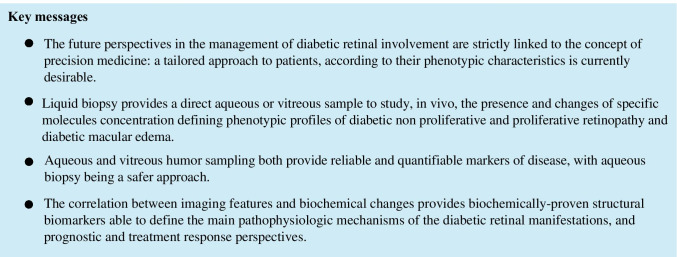

## Introduction

Diabetic retinopathy (DR) represents one of the leading causes of visual impairment and preventable blindness worldwide [1]. It is estimated that around 425 million people are affected by diabetes and around 212 million are undiagnosed subjects. It has also been estimated that approximately 93 million people worldwide have DR and that one-third of the global diabetic population is expected to develop some degree of retinopathy during lifetime [1, 2]. A detailed analysis of these data highlights that the main sight-threatening complications of diabetes, namely, diabetic macular edema (DME) and proliferative DR (PDR), will represent an increasing burden for the diabetic population in the near future and a serious socio-economic health problem [3–5].

The high impact of ocular diabetic complications increases the importance of searching new approaches to better understand the exact pathophysiology of human DR since its very early phases. It seems essential to be able to quantitatively delineate the fine intraocular mechanisms inducing the development and step-by-step progression of DR. In the past, DR was identified by ophthalmoscopy and then documented by fundus photography. Fundus fluorescein angiography helped to understand the microvasculature component of this disorder, confirming previous histopathologic studies performed both in diabetic animals and enucleated human eyes. Optical coherence tomography (OCT) allowed to visualize and understand the intraretinal—layer-by-layer—alterations (not only microvascular) induced by diabetes mellitus in the human retina and choroid. Using these diagnostic approaches, it has been possible to better identify different retinal (and choroidal) parameters (i.e., location and extent of macular edema, sub-retinal fluid, disorganization up to atrophy of single or multiple retinal layers, location and extension of both macular, and peripheral ischemia) useful to address therapy. One of the main biochemical pathway implicated in the development and progression of DR is related to the vascular endothelial growth factor (VEGF) family, mainly VEGF-A [6–8]. The inhibition of VEGF has been considered the best approach to treat pharmacologically both DME and PDR and to prevent progression (or induce regression) of DR. Unfortunately, clinical evidence has shown that intravitreal anti-VEGF therapy is able to reduce DME in about 60% of cases and, on a long-term perspective, panretinal photocoagulation remains the gold standard in the treatment of PDR [9, 10]. This probably means that even current sophisticated imaging technologies are unable to detect and differentiate the hypothesized human phenotypes of DR [11, 12]. This fact has recently induced clinical researchers to try to quantify the exact intraocular molecular changes induced by diabetes into the retina. This approach has opened the way to “liquid biopsy” to enter in the retina scenario of diabetes research. The concept of “liquid biopsy” has emerged as a general approach in medicine and was introduced by the medical oncology disciplines. This diagnostic approach in oncology aims at integrating information from liquid samples, namely, blood, to provide precise and detailed information about tumor progression [13]. Liquid biopsy appears to be a crucial complement to the more invasive tissue biopsy, both for diagnosis and management of cancer [14]. This approach is also relevant to provide “phenotypic” information of a specific disease. Therefore, liquid biopsy has become a routinely approach performed not only in oncology, but also in other medical branches, such as neurology and rheumatology [15–17]. This diagnostic approach, which has dramatically changed the clinical practice in other medical specialties, should be applied in ophthalmology too. This may be obtained by sampling and analyzing ocular fluids, in particular aqueous or vitreous humor, because proteomic and metabolomic analysis of these ocular fluids has the potential to add new information about the pathophysiology and monitoring of ocular disorders, even at the retinal level [14].

In humans, the most readily accessible ocular tissues are tears and ocular surface components such as the cornea and conjunctiva. These ocular matrices may provide valuable information regarding anterior segment disorders and have also been studied in DR. However, it is the aqueous humor (AH) and vitreous which are more suitable matrices for the evaluation of relevant biomarkers for posterior segment disorders [18].

This article reviews current results and promising perspectives opened by liquid biopsy on intraocular fluids, namely, aqueous and vitreous humor, in DR.

## Methods

To identify potentially relevant articles in the medical literature, we searched MEDLINE for English language articles published from January 1980 to December 2020. MEDLINE was queried using the following search terms (used both individually and in combination for advanced research): proteome, proteomics, metabolome, metabolomics, biomarker, eye, retina, diabetic macular edema, and diabetic retinopathy. Additional articles were identified by reviewing the references of examined publications. To identify potentially relevant articles to be included in this review, two investigators reviewed each paper. Case series were preferred to single-case reports. Articles included in the reference list were fully examined by the authors.

## Intraocular sampling

The foundations for understanding the pathophysiology of ocular, and particularly retinal, diseases have been built up in animal studies, because of the intrinsic limitations of a direct histological and molecular human eye examination [19]. However, it is mandatory to confirm animal experimental data in humans, for example, searching eye disease biomarkers into the accessible human compartments [15]. At present, liquid biopsy may be applied to the eye compartments, in particular vitreous and aqueous, for a more direct comprehension and phenotyping of intraocular disorders, including the retinal ones [15, 16]. It may be questioned if AH analysis may reliably reflect a retinal condition, as vitreous humor does. However, some authors have demonstrated a full correlation of the molecular content in simultaneous aqueous and vitreous samples, from the same eye [20–22]. Funatsu et al. specifically planned a study where aqueous and vitreous of the same eye were contemporarily sampled to quantify specific intraocular cytokine levels [20]. VEGF and IL-6 levels in aqueous and vitreous humors were significantly higher than the plasma levels and significantly correlated with each other and with the severity of diabetic retinopathy [20]. Therefore, AH proteins concentration may be clinically useful as the vitreous one [20, 23]. This data, confirmed by other authors in different posterior segment disorders, represents a milestone in the “liquid biopsy approach” to retinal disorders [21, 22].

### Biodynamic of ocular fluids: posterior to anterior route

Juneman et al. proved the passage from the retina to the vitreous of a retinal glial activation–related protein: the glial fibrillary acidic protein (GFAP). GFAP is typically produced by astrocytes in healthy conditions and by other retinal cells, such as activated Müller cells, in diabetes [24]. In diabetic retinopathy, Müller cells show morphologic changes, such as hypertrophy, associated with GFAP production [25]. This is a remarkably ubiquitous response that can be observed in some forms of retinal stress, damage, and degeneration, including retinal detachment and retinal photocoagulation [26, 27]. Moreover, the presence of GFAP in body fluids has already been reported and proposed as a biomarker of glial activation and pathology in neurological diseases [27, 28]. It has been reported that the modifications and alteration of Müller cell triggers cellular proteolysis. With proteolytic break-up of the GFAP polymer, soluble fragments of GFAP are released to the adjacent fluid compartments. Therefore, GFAP might be used as an indirect marker for Müller cell activation, protease activation, and eventually secondary degenerative processes in the retina [24]. Other experimental studies on the biodynamic of ocular fluids were instrumental to elucidate the mechanism through which GFAP—or any other molecule produced and released by the retina—may reach, through the vitreous, the anterior chamber [29, 30]. Maurice et al. reported in detail about the flow of intraocular fluids from vitreous to aqueous, in animal models [30]. They demonstrated, using a thermal diffusional analogue, that the passage of molecules out of the vitreous is entirely through the anterior chamber and that it is characterized by a slow diffusion within the anterior vitreous humor [30]. A vitreous-to-aqueous gradient has also been demonstrated in humans, promoting the anterior diffusion of VEGF and other angiogenic factors, potentially accounting for the occurrence of anterior segment neovascularization in association with wide retinal ischemia. This gradient may be due to the rapid clearance of proteins from the anterior chamber or their more rapid degradation in the same location [19, 31]. The molecular weight of GFAP (around 50 kDA) is very similar to the proteins quantified by Maurice et al.: thus, the passage of GFAP, from the retina through the vitreous into the anterior chamber, is clearly possible [30]. More recently, studies about intraocular fluid dynamics aimed at clarifying the pharmacokinetic of drug delivery systems have shown that, after an intravitreal injection, the drug is eliminated from the eye, either via the anterior route (anterior chamber) or through the retina [29, 32]. The anterior route is free for all drugs which enters the anterior chamber and then are eliminated via the aqueous compartment outflow [29]. It is exactly the settling of molecules in the anterior chamber that justifies sampling AH to make a proteomic and/or metabolomic analysis in retinal disorders. Therefore, intraocular fluid biodynamic supports the concept that sampling and analyzing the protein content in the AH represent another safer, reasonable way to study a posterior segment condition, mainly because vitreous sampling is a more invasive procedure.

### Safety

The use of AH paracentesis as diagnostic procedure in several ocular diseases, such as uveitis, but also as therapeutic option, for example, in acute primary angle-closure glaucoma, has proved to be a safe and effective technique in several reports. In acute pathologic conditions, different factors may complicate this procedure, such as corneal edema, high intraocular pressure, flare, and acute eye inflammation. However, even in these conditions, no safety issues have been reported, even if AH sampling is performed at the slit lamp [33, 34]. In our literature research, no significant adverse event was reported following AH biopsy, even in phakic patients, regardless of patients’ age. However, the recommendations for a safe procedure include the use of adequate topical anesthesia and disinfection (povidone iodine), of eyelid speculum and drape, preferably performed in an operating room, before or separately from any other surgical procedure, including intravitreal injection [33, 35]. Conversely, vitreous biopsy may raise some greater concerns in terms of safety, compared to AH biopsy, because of the intrinsic greater invasiveness of the procedure. Therefore, it is quite exclusively performed before or during a surgical procedure for a different clinical indication, such as vitrectomy for PDR or epiretinal membrane (as later discussed). However, both these procedures, when correctly performed, have proved to be safe also in eyes affected by tumoral lesions with risk of metastases, such as uveal melanoma. As already mentioned, one of the most important field of application of liquid biopsy is oncology. A growing amount of studies are showing the relevance of this approach also in ocular oncology, not only to directly study the tumoral lesions but also to analyze the tumor-related microenvironment changes, detectable in ocular fluids [36–38].

Finally, another critical point when discussing about intraocular sampling to quantify molecular biomarkers of retinal and choroidal disorders is the control group. It is commonly agreed that healthy controls are represented by a group of age-matched subjects, unaffected by concomitant relevant systemic or ocular disorders, which may act as confounders of biomarker quantification. The invasiveness of vitreous sampling may raise ethical concerns, thus restricting the possible control group to organ donors or patients undergoing vitrectomy for non-DR-related diseases, such as macular hole, epiretinal membrane, or retinal detachment [24, 39]. For intraocular AH biomarkers studies, the control subjects are commonly chosen among healthy people undergoing an already planned cataract surgery, and the AH sample is obtained as first step of the surgical procedure. Yao J et al. confirmed the reliability of these controls, and this approach is now widely accepted in studies of intraocular proteomics [40–46].

## Proteomics and metabolomics in diabetic retina

### Proteomics

Human fluids are represented by a complex mixture of cells, electrolytes, organic solutes, and proteins of different molecular weight, such as growth factors, cytokines, and additional proteins whose main function is to provide the metabolic requirements to the ocular tissues. The identification and quantification of proteins, including their isoforms, variants, and posttranslational modifications, in the compartments of the eye in both health and disease are addressed as “proteomics” of intraocular fluids [47].

The National Institutes of Health Biomarkers Definitions Working Group has defined a biomarker as “a characteristic that is objectively measured and evaluated as an indicator of normal biological processes, pathogenic processes, or pharmacologic responses to a therapeutic intervention” [48]. Regarding the eye, both imaging and biochemical biomarkers may be considered. Circulating (serum) biochemical biomarkers have poorly contributed to the comprehension and management of DR and diabetic maculopathy and are not applied in clinical practice. This is mainly because of the limited number of correlations found between serum parameters (mainly inflammatory cytokines, such as interleukins and VEGF) and diabetic-related retinal complications (such as DME, PDR, or foveal avascular zone enlargement) [15, 49]. As regards imaging biomarkers, the use of structural OCT has certainly contributed to quantify some retinal parameters, such as central retinal thickness and the presence and characteristics of intra-retinal or sub-retinal fluid. A new promising diagnostic technology is OCT angiography, but its full validation in DR is still under debate [50]. Conversely, direct ocular sampling—vitreous and/or aqueous—has the potential to offer more detailed information, which may be defined more “quantitative” than “qualitative,” through the detection of biochemical local biomarkers. In fact, the quantification of specific protein concentration and its variation in different disease phases (compared to controls) provide a precise tool to define each eye condition. Several proteomic studies have been published about posterior segment disorders, such as DR and DME [29, 32, 41, 42].

### Metabolomics

In the very recent years, a newest approach, the metabolomics, has also been developed both in vitreous and aqueous humor samples to evaluate retinal disorders, DR in particular [51]. The “metabolome” represents a set of metabolites in a biological tissue, in this case the eye, which are the end-products of a specific cellular process [18, 51, 52]. Metabolomics specifically seek to measure those metabolites which change in response to a stimulus of one sort or another, providing a dynamic picture of the processes occurring into the eye. For example, the recent AH metabolomic studies on diabetic patients showed a possible alteration of mitochondrial function in long-duration diabetics and oxidative stress and endothelial damage. This new approach may add novel insights in the altered biological processes of the retina, coupling biochemical information to clinical ones [18, 51–55].

## Liquid biopsy results in diabetic retinopathy

In the last decades, the rapid advancement in biotechnology, engineering, and equipment has led to the possibility to dose, even in small amounts of ocular samples (vitreous and aqueous), a high number of molecules, which might be related to the presence of DR, PDR, and DME, and their progression. The main studies on vitreous and aqueous samples are summarized hereafter.

### Proliferative diabetic retinopathy

Studies on vitreous samples of DR eyes have primarily been performed on the proliferative stage of DR (PDR), during a planned vitrectomy, for therapeutic aims [39]. These studies have confirmed the role, in the pathogenesis of new vessel growth and proliferation, not only of VEGF, but also of several inflammatory factors [56–70]. The vitreous levels of VEGF and IL-6 were positively correlated with the clinical grade of PDR, particularly in its active stage, defined by a significant amount of perfused preretinal new capillaries [20]. Moreover, a systematic meta-analysis of biomarkers investigated in the vitreous of diabetic patients has shown the presence of around 11 molecules as possible new targets for potential treatment, beyond the already known anti-VEGF drugs. Four of them have been deemed viable targets for PDR: eritropoietin A and B receptors, anti-platelet-derived growth factor-BB, and pigment epithelium-derived factor [39]. Some authors have recently identified other potential biomarkers of PDR, such as macrophage migration inhibitory factor (MIF), previously found to be upregulated in animal models of corneal neovascularization. It has been detected in endothelial cells, leukocytes, and myofibroblasts in epiretinal fibrovascular membranes from patients with PDR, as well as in their vitreous fluid. It causes the upregulation of VEGF in Müller cells, leading to angiogenesis and thus representing a new possible target for treatment [71]. Balaiya et al. studied patients with PDR, by sampling both aqueous and vitreous, and showed an increase of several molecules, representing the different mechanisms involved in DR progression. The detection of fibrinogen molecules, as well as alpha-2 macroglobulin in PDR vitreous, confirmed the relevance of a hypercoagulable state in the pathogenesis of diabetes-related severe damage [72–74]. Moreover, they suggested that different vitreous levels of antithrombin III, an inhibitor of coagulation, may be related to different clinical stages of PDR (vasoproliferative vs fibrotic). The increase, in PDR vitreous, of factors belonging to complement and kallikrein–kinin systems, responsible for severe ocular inflammation, and involved into the progression to proliferative forms of DR, confirmed the inflammatory mediators as therapeutic targets for the advanced stages of PDR [72, 75, 76]. The dysregulation of vitreous levels of the proinflammatory and proangiogenic factor osteoprotegerin and its ligands was also detected in PDR eyes [77]. Inflammation, hypoxia, and oxidative stress also stimulate the production, activation, and signaling functions of matrix metalloproteinases, which are increased in the vitreous humor of PDR patients. They are involved in angiogenesis, loss of photoreceptors, and blood–retina barrier breakdown, thus appearing as disease biomarkers and targets for therapeutic inhibitors [78, 79]. Furthermore, other studies have compared the concentration of VEGF before and after vitrectomy, showing that, in the majority of patients, the level of VEGF was significantly and successfully reduced and suggesting that those with a high permanence of VEGF levels after vitrectomy are more prone to ocular complications such as neovascular glaucoma and that the ratio of remnant VEGF and pre-operative VEGF concentration may represent a predictor of late complications [80–84].

### Preclinical and clinical non-proliferative diabetic retinopathy

In diabetic patients with early or preclinical stages of DR, who do not need vitrectomy, vitreous sampling may represent an unjustified procedure, while AH sampling proved to be more applicable, equally reliable, and meaningful.

Preclinical stages of DR have been investigated, in order to detect the very early drivers of retinopathy [85–93]. Chiang SY et al., for example, compared diabetic patients without and with clinical signs of DR and demonstrated increased levels of total protein in the AH but also a different AH protein profile in DR patients [85]. Factors involved in nutrition transport (apolipoprotein A-I, serotransferrin), microstructure reorganization [keratin type I cytoskeletal 9 (KRT9), keratin type I cytoskeletal 10 (KRT10), podocan (PODN)], and neuroprotection [cystathionine beta-synthase (CBS)] were hyperexpressed in DR eyes, and angiogenesis-related factors [growth factor receptor-bound protein 10 (GRB10), brain-specific angiogenesis inhibitor 1-associated protein 2 (BAIAP2)] were detected only in AH of DR patients [85]. Moreover, a significant difference in the concentration of several cytokines and chemokines involved in inflammation and angiogenesis was detected in the AH of diabetic patients compared to controls, increasing with DR severity [higher levels of interleukin (IL)-1β, Il-6, and Il-8, monocyte chemo-attractant protein (MCP)-1, interferon gamma-induced protein-10, VEGF, and reduced levels of IL-10 and Il-12] [94]. The critical role of inflammation in the development of DR has been firstly described in vitro and in animal models: glutamate, proteases, leukotrienes, IL-1β, IL-6, TNF-α, VEGF, lymphotoxin MMPs, and ROS were linked to DR [95]. Moreover, glial cells, particularly microglia and Müller cells, have proven to have an initial role in the inflammation pathway [95]. Other in vivo studies have confirmed the early activation of the inflammatory processes secondary to chronic hyperglycemia, using AH samples [42, 96]. Moreover, they demonstrated, in vivo, retinal macroglial cell activation, by the detection of specific cellular biomarkers. GFAP, aquaporin (AQP)1, and AQP4—biomarkers of Müller cell activity—showed to be significantly increased in human eyes with diabetes, confirming that glial cells are precociously affected by diabetes mellitus. In particular, GFAP and AQP4 levels were higher also in diabetic eyes without clinical signs of DR, and they have been suggested as early biomarkers of diabetes-induced retinal stress [42].

### Diabetic macular edema and response to treatment

Diabetic macular edema is one of the most important retinal complications of diabetes, requiring early and adequate intervention to limit a rapid functional deterioration. It results from the dysregulation of the complex interactions between neuronal degeneration, retinal inflammation, macroglial dysfunction, and microvascular damage, leading to chronic intraretinal fluid accumulation [97, 98]. As for non-proliferative DR, DME eyes, not requiring vitrectomy, were mainly studied by sampling AH. In these eyes, a further increase of some factors/cytokines [VEGF, IL-6, IL-8, interferon inducible protein (IP)-10, leukemia inhibitor factor (LIF), HGF hepatocyte growth factor, VEGF vascular, intercellular adhesion molecule-1 (ICAM-1), platelet-derived growth factor (PDGF)] was found, compared not only to non-DR eyes but also to DR eyes without DME [99–102]. In particular, ICAM-1, which is known to potentiate retinal vascular leukocyte adhesion, increase vascular permeability, and promote capillary closure in response to elevated ambient VEGF levels, was identified as a biomarker for disease severity [101]. The interest for inflammation as main driver of DR progression and DME onset has led to the dramatic rise of studies demonstrating an increase of inflammation-related factors in the AH samples of diabetic subjects with DR and/or DME [29, 32, 99–101, 103–108]. A higher concentration of specific Müller cell–related factors has been reported, namely, GFAP and inwardly rectifying potassium channel (Kir) 4.1, which in vitro and animal studies have already demonstrated to be overexpressed and altered in distribution, secondary to Müller cell activation [96]. Inflammatory factors were also found in the AH of patients affected by macular edema of different origin [109, 110]. Chu et al. found that the concentration of some inflammatory cytokines, such as IL-1β, IL-6, MCP-1, IL-10, and VEGF, was correlated to macular thickness in post-cataract surgery patients, thus suggesting them as potential predictors of postoperative macular thickening [109].

Prognosis and response to treatment are two of the main points potentially addressed by the liquid biopsy approach. Recently, different studies have reported the response to DME treatment, by means of aqueous and vitreous samples [32, 104, 105, 111–116]. Sohn et al. compared the AH concentration of VEGF and inflammatory molecules after the administration of triamcinolone and bevacizumab, showing that the first one was able to reduce several inflammatory molecules (IL-6, IP-10, MCP-1, PDGF-AA) and VEGF concentration, while bevacizumab just reduced VEGF concentration [32]. These data confirm the multifactorial pathophysiology of DME, not only related to VEGF, but also to a wider inflammatory activation [32].

The use of serial AH sampling has also elucidated the effects and mechanisms of action of other DME treatments, such as subthreshold micropulse laser (SMPL). At 1-year follow-up, repeated successful SMPL treatments (in terms of reduced central retinal thickness and improved visual acuity), caused a reduction of several retinal molecules, including VEGF and other inflammatory ones [117]. Moreover, the biomarkers of Müller cell activation—GFAP and Kir 4.1—were significantly reduced after SMPL, showing a sort of de-activation and normalization of the retinal environment, and especially a restoration of Müller cell function [90]. No significant changes of the retinal pigment epithelium (RPE)–related biomarkers were found after SMPL treatment, suggesting that RPE might not represent the main target of this laser technology [118]. These data may represent a new perspective in the comprehension of the mechanisms of DME therapies, such as SMPL, and a new way for its possible application, even in different retinal disorders.

### Future perspectives

Liquid biopsy has dramatically renewed the approach to patients in oncology, introducing the concept of patient-tailored medicine, thus significantly improving not only our knowledge about neoplastic diseases, but also the individual management of cancer patients [119]. In ophthalmology, this approach has proved to be able to detect repeatable and quantifiable biomarkers involved in fluid homeostasis and its changes secondary to eye diseases, as DR. And the concept of disease “phenotype” has assumed a greater role in the definition of retinal diseases [16, 120, 121]. The detection of different structural profiles, particularly as concerns macular edema, opened the possibility to identify patients with different patterns and timing of disease onset and evolution, as well as response to therapy. The development of high-technology retinal imaging techniques has allowed the identification of a series of structural biomarkers which are still extensively under study to find a reliable correlation with the pathophysiological and functional aspects [122]. Some imaging biomarkers, such as sub-retinal fluid and hyperreflective intraretinal foci, have proved to be highly related to visual acuity and retinal sensitivity as well as to the morphologic and functional response to therapy, not only in DME but also in other retinal diseases, such as age-related macular degeneration and retinal vein occlusion [121, 123]. However, the main limitation of these imaging biomarkers is their indirect nature, allowing only for a statistics- and experience-related interpretation. Therefore, the possibility to correlate imaging and biochemical biomarkers may provide a direct link between a specific imaging biomarker to a pathophysiologic mechanism, identified by a proteomic and metabolomic profile. Noma et al., for example, detected a higher concentration of IL-6 and VEGF in aqueous and vitreous samples of patients affected by retinal vein occlusion with serous retinal detachment, directly correlating the increased vascular permeability related to these factors to the presence of the imaging biomarker “sub-retinal fluid” [124]. In the future, the possibility to correlate imaging and biochemical biomarkers will overcome the current limitations of imaging, providing biochemically proven structural biomarkers able to easily and non-invasively detect specific pathophysiologic retinal processes. Changes in intraocular fluids proteomics and metabolomics have already proved to correlate with the prognosis of eye disorders [16]. The application of liquid biopsy to the structural retinal analysis may allow a broad application to a wide range of settings, from diagnosis (including screening) prognosis and the prediction of response or resistance to treatments. Moreover, it would allow a personalized approach to each patient, both in terms of therapeutic choice and follow-up timing.

## Conclusions

The future management of chronic eye disorders will be strictly linked to the concept of precision medicine, requiring a tailored approach to patients, according to their phenotypic characteristics. In this perspective, sampling directly the eye, by means of liquid biopsy, including either aqueous or vitreous sampling, allows to obtain a direct biologic sample to study, in vivo, the presence and changes of specific molecules concentration. This approach seems to overcome the limitations of imaging biomarkers, even the most advanced, such as OCT angiography. This is due to the possibility, offered by liquid biopsy, to analyze a specimen obtained in vivo, which may clarify which process is responsible for a specific phenotype. Therefore, the liquid biopsy approach, as already proven in other medical specialties, represents, to date, the most reasonable and accurate way to offer, also in ophthalmology, a completely new insight in retinal disorders, as DR.

DR still represents one of the retinal disorders with the most severe socio-economic impact, increasing even in the young population. Considering the relevance of this disease, it’s not surprising that it represents the main field of application of liquid biopsy in ophthalmology. In fact, liquid biopsy has the potential to dramatically modify the knowledge of this retinal disorder and also provide new and innovative hypothesis for treatment strategies, opening the way to an individualized approach, based on the detection of specific biomarkers related to biochemically proven pathogenetic retinal processes. In the future, liquid biopsy may be extended to study other retinal disorders, such as age-related macular degeneration and those retinal diseases whose pathophysiology remains mostly unknown.

## Data Availability

All authors had full access to the material used for this review.
